# Type B Insulin Resistance Masquerading as Ovarian Hyperthecosis

**DOI:** 10.1210/jc.2016-3674

**Published:** 2016-12-02

**Authors:** Rebecca J. Brown, Jalaja Joseph, Elaine Cochran, Cornelia Gewert, Robert Semple, Phillip Gorden

**Affiliations:** 1 National Institute of Diabetes and Digestive and Kidney Diseases, National Institutes of Health, Bethesda, Maryland 20892; 2 The University of Cambridge Metabolic Research Laboratories, Wellcome Trust-MRC Institute of Metabolic Science, Cambridge CB2 OQQ, United Kingdom; 3 The National Institute for Health Research Cambridge Biomedical Research Centre, Cambridge CB2 OQQ, United Kingdom

## Abstract

**Context:**

Hyperinsulinemia can lead to pathologic ovarian growth and androgen production.

**Case Description:**

A 29-year-old woman developed an autoantibody to the insulin receptor (type B insulin resistance), causing extreme insulin resistance and hyperinsulinemia. Testosterone levels were elevated to the adult male range. Treatment with gonadotropin-releasing hormone (GnRH) analog led to normalization of testosterone, despite persistent extreme insulin resistance.

**Conclusions:**

This case demonstrates that gonadotropins are necessary for insulin to cause pathologic ovarian androgen production. Suppression of gonadotropins with GnRH analogs may be a useful therapeutic option in patients with severe hyperandrogenism or ovarian enlargement because of hyperinsulinemia.

## Teaser

We studied a patient whose case demonstrated that gonadotropins are required as cofactors for insulin-induced hyperandrogenism in type B insulin resistance.

Severe hyperandrogenism causing virilization in women is rare; causes include congenital adrenal hyperplasia, adrenal or ovarian tumors, Cushing syndrome, and ovarian hyperthecosis. Ovarian hyperthecosis is a rare disorder of severe, functional ovarian hyperandrogenism, usually associated with insulin resistance (IR), similar to polycystic ovarian syndrome (PCOS). Extreme forms of IR, including lipodystrophy, mutations of the insulin receptor, or autoantibodies to the insulin receptor (type B IR), represent even more dramatic examples of IR leading to functional ovarian hyperandrogenism, and may be associated with massive ovarian enlargement and testosterone levels in the adult male range (1).

It was previously suggested that, in extreme IR, insulin alone could lead to pathologic ovarian androgen production, independent of gonadotropins (1). Here, we present a case demonstrating that gonadotropins are required as cofactors for insulin-induced hyperandrogenism in type B IR.

## Case Presentation

A previously healthy 29-year-old African American woman developed secondary amenorrhea, followed 8 months later by polyuria, polydipsia, and 20-lb (9.1 kg) weight loss. Blood glucose was 40 to 400 mg/dL; hemoglobin A1c was 6.1%. She had symptoms of virilization, including deepened voice, decreased breast size, android body shape, acne, clitoromegaly, hirsutism, and increased rage. Darkening of the skin occurred on the face, axillae, elbows, and abdomen. Laboratory evaluation revealed markedly elevated total and free testosterone [total: 450 to 610 ng/dL (normal: 2 to 45 ng/dL), free: 25.6 pg/mL (normal: 0.2 to 5 pg/mL)]. Adrenal androgens were normal [17-hydroxyprogesterone: 102 ng/dL (normal <185 ng/dL), dehydroepiandrosterone sulfate: 84 µg/dL (normal: 40 to 325 µg/dL)]. Gonadotropins were normal [luteinizing hormone (LH): 13.7 IU/mL, follicle-stimulating hormone: 5.1 IU/mL]. Insulin-like growth factor 1 was 100 ng/mL (normal: 117 to 329 ng/mL). Mild pancytopenia was noted. Imaging showed bilaterally enlarged ovaries with numerous follicles consistent with PCOS, without masses; the adrenals appeared normal. Because of the severity of the testosterone elevation, an ovarian tumor was suspected despite these imaging results. Therefore, ovarian venous sampling was performed, which showed testosterone >1500 ng/dL bilaterally. The patient received a presumptive diagnosis of ovarian hyperthecosis; leuprolide acetate depot injection 22.5 mg intramuscularly was administered.

**Figure 1.**
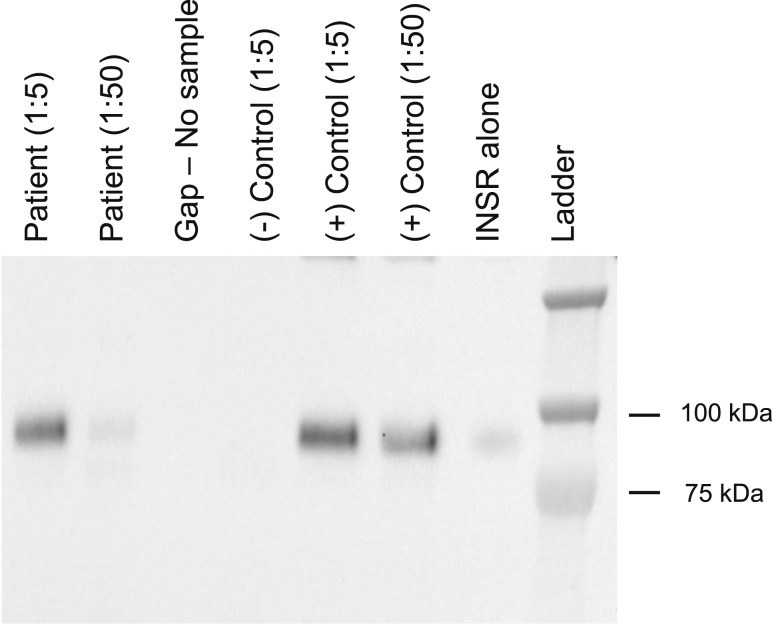
Anti-insulin receptor autoantibody assay. Anti-insulin receptor autoantibodies are demonstrated by immunoprecipitation of solubilized insulin receptors with, from left to right: the patient’s serum at 1:5 and 1:50 dilutions, compared with negative (1:5 dilution) and positive (1:5 and 1:50 dilutions) controls, and the INSR alone (10). For detection of endogenous anti-insulin receptor antibodies, serum was first diluted 1 in 5 or 1 in 50 in phosphate-buffered saline prior to incubation with an optimized concentration of a crude preparation of recombinant human INSR (hINSR; a lysate of CHO cells stably expressing human insulin receptor) in immunoprecipitation buffer (2.52 g/L NaF, 8.92 g/L Na_4_P_2_O_7_, 100 mM HEPES, and 300 mM NaCl) for 4 hours at 2°C to 8°C with gentle agitation. Antibodies were then captured using goat antihuman IgG agarose beads (A3316, Sigma, Billerica, MA; 2 hours at 2-8°C with gentle agitation). Unbound hINSR was washed away with bead wash buffer (immunoprecipitation buffer as previously mentioned with the addition of 10 mM EDTA, pH 8.0, and 0.2% Triton-X 100) before reducing and fragmenting captured hINSR using Laemmli buffer. Samples were resolved by sodium dodecyl sulfate–polyacrylamide gel electrophoresis on 8% Bis-Tris gels before detection of hINSR beta subunit using hINSR beta subunit-specific antibody (sc-711; Santa Cruz, Dallas, TX) by immunoblotting. INSR, insulin receptor.

Three months after the leuprolide, the patient was evaluated at the National Institutes of Health after signing informed consent under a natural history study of disorders of IR (ClinicalTrials.gov no. NCT00001987), approved by the Institutional Review Board of the National Institute of Diabetes and Digestive and Kidney Diseases. She reported improved mood and skin tone, normal blood glucose except for occasional fasting hypoglycemia, weight gain, and regression of clitoromegaly. Examination revealed hirsutism and mild acanthosis nigricans in the neck and malar distribution. Testosterone was <20 ng/dL, LH was 0.4 U/L, follicle-stimulating hormone was 2.1 U/L, and fasting insulin was 29.3 µU/mL. Serum antibodies against the insulin receptor were present (Fig. 1), confirming the diagnosis of type B IR. Because the patient appeared to be entering spontaneous remission, no treatment was given; it was not clear whether her low testosterone was attributable to her remission or leuprolide.

At age 33 years, the patient returned with hyperglycemia and hyperandrogenic symptoms. Hemoglobin A1c was 4.3% (falsely low because of active hemolysis), fasting insulin was 279.6 µU/mL, and glucose was 122 mg/dL. Anti-Smith/ribonucleoprotein antibody was >200 IU (normal: <20), anti-nuclear antibody was >12 IU (normal: 0 to 0.9 IU), anti-extractable nuclear antigens screen was positive at >200 IU (normal: 0 to 19 IU). Pancytopenia was noted. Testosterone was elevated at 777 ng/dL, with free testosterone at 7.4 ng/dL (normal: 0.1 to 2.4 ng/dL). The patient elected to defer immunosuppression for treatment of type B IR, and instead received depot leuprolide acetate. Two months later, total testosterone was 33.8 ng/dL (free testosterone: 0.2 ng/dL). However, blood glucose was still elevated at 182 mg/dL, and fasting insulin was 143.7 µU/mL. Antibody depletion therapy with rituximab, pulsed dexamethasone, and oral cyclophosphamide was initiated as previously described (2). Five months later, diabetes and IR had resolved, menstrual periods had resumed, and total testosterone was 45 ng/dL (normal: <81 ng/dL).

## Discussion

In this patient, a fortuitous missed diagnosis led to the observation that gonadotropin suppression with gonadotropin-releasing hormone (GnRH) analog could effectively treat the hyperandrogenism (but not the diabetes) of type B IR. Although antibody depletion therapy addresses the root cause of type B IR, thus ameliorating both hyperandrogenism and diabetes (2), GnRH analogs may be an alternative approach for patients who have contraindications to immunosuppressive drugs, or are unwilling to accept their side effects. Moreover, GnRH analogs may be options for women with severe hyperandrogenism caused by insulin receptor mutations, who might otherwise require oophorectomy.

In 1975, Flier *et al.* (3) reported that, in 3 of 6 patients with extreme IR, a serum factor (thought to be an antibody) was present that impaired insulin binding to its receptor. The waxing and waning nature of type B IR, with spontaneous remissions and recurrences, and hyper- and hypoglycemic manifestations, was described in 1978 (4).

Hyperandrogenism was not in the initial descriptions of type B IR, but it was later recognized as a frequent feature (5). Although gonadotropins serve as normal physiologic stimuli for ovarian growth and steroidogenesis after puberty, insulin can act as a pathologic growth factor and stimulator of sex steroid production. The correlation between hyperinsulinemia and hyperandrogenemia in women with PCOS was first noted in 1980, but the direction of causality was not clear (6). The mechanisms by which hyperinsulinemia leads to androgen excess are complex (7). Both insulin and IGF-1 receptors are present in ovary, but most of insulin’s effects on steroidogenesis appear to be mediated through the insulin receptor. Insulin synergizes with LH to upregulate CYP17, thereby increasing testosterone production (7). In typical PCOS, there is selective resistance to insulin downstream of the insulin receptor in pathways regulating glucose, whereas sensitivity to insulin’s effects to increase steroidogenesis in granulosa and theca cells is maintained (7). In patients with dysfunctional insulin receptors, including type B IR or insulin receptor mutations, it is not clear how insulin signals within the ovary to increase androgen production because one would expect all pathways downstream of the insulin receptor to be blocked. Furthermore, in rodents, selective insulin receptor deletion in theca cells prevents hyperandrogenism induced by hyperinsulinemia (8); however, germline insulin receptor mutations in humans do result in hyperandrogenism (9). It is possible that insulin signals through hybrid insulin/IGF-1 receptors. Regardless of this confusion, studies of patients with type B IR strongly support a causal role for insulin in hyperandrogenism because testosterone increases when insulin is elevated, and normalizes when remission occurs and insulinemia returns to normal (5).

A previous publication by our group suggested that insulin was sufficient to cause pathologic ovarian enlargement and steroidogenesis, independent of gonadotropins (1). This conclusion was based on 4 patients with extreme IR who had LH ≤1 U/L, with either ovarian enlargement, elevated testosterone, or both. Based on these observations, it was thought that suppressing gonadotropins pharmacologically in patients with extreme IR would not resolve insulin-induced hyperandrogenism. On reviewing these cases, however, the 2 of these 4 patients who had testosterone levels >2 standard deviations above normal were clinically in puberty based on breast development. Similarly, of 18 postpubertal women with type B IR, 2 had elevated testosterone despite LH ≤1 U/L (unpublished data). Thus, although these patients’ random LH levels were undetectable, they presumably had sufficient LH pulsatility to stimulate ovarian steroidogenesis. The current case demonstrates that, similar to other disorders of excess ovarian androgen synthesis, insulin-induced ovarian androgen excess requires the presence of gonadotropins, and can be suppressed by GnRH agonists.
